# A protein phosphatase 2A deficit in the hippocampal CA1 area impairs memory extinction

**DOI:** 10.1186/s13041-019-0469-9

**Published:** 2019-05-21

**Authors:** Jing Wang, Ran Xie, Xiaolin Kou, Yu Liu, Cui Qi, Rui Liu, Weiyan You, Jun Gao, Xiang Gao

**Affiliations:** 1 Department of Neurobiology, Nanjing Medical University, Nanjing, 211166 Jiangsu China; 2 Model Animal Research Center and MOE Key Laboratory of Model Animals for Disease Study, Nanjing University, Nanjing, 210093 Jiangsu China

**Keywords:** PP2A, Extinction, Learning and memory, Synaptic plasticity, Hippocampus

## Abstract

Protein phosphorylation plays an important role in learning and memory. Protein phosphatase 2A (PP2A) is a serine/threonine phosphatase involved in the regulation of neural synaptic plasticity. Here, to determine if PP2A is necessary for successful learning and memory, we have utilized a *Tg (Camk2a-cre) T29–2Stl* mice to specific knock down the expression of hippocampal PP2A in mice. By analysing behavioural, we observed that loss of PP2A in the hippocampal CA1 area did not affect the formation of memory but impaired contextual fear memory extinction. We use the electrophysiological recording to find the synaptic mechanisms. The results showed that the basic synapse transmission and synaptic plasticity of PP2A conditional knockout (CKO) mice were impaired. Moreover, PP2A CKO mice exhibited a saturating long-term potentiation inducted by strong theta burst stimulation but no depotentiation after low-frequency stimulation. Taken together, our results provide the evidence that PP2A is involved in synaptic transmission and hippocampus-dependent memory extinction.

## Introduction

The higher-order brain functions have been the subjects of intense research in the neurosciences over the past decades. And the major brain functions, including learning and memory, rely on brain plasticity and involve changes in synaptic plasticity. The mechanisms that underlie synaptic plasticity in the brain are complex and depend on multiple cascades of molecular events. Research on the mechanism of different phases of learning and memory has been underway for many years. Phosphorylation and dephosphorylation of proteins are the critical regulatory mechanism that underlies proper learning and memory and synaptic plasticity. Protein kinases and protein phosphatases are important players in the induction of both long-term potentiation (LTP) and long-term depression (LTD). Serine/threonine phosphatases play an important role in synaptic plasticity by regulating the phosphorylation state of key proteins. There are three major families of serine/threonine phosphatases: phosphoprotein phosphatases (PPPs), metal-dependent protein phosphatases, and aspartate-based phosphatases [1]. Protein phosphatase 1 (PP1), 2A (PP2A), and 2B (PP2B) are belong to PPPs, and numerous studies have established that they are involved in synaptic plasticity. Evidence from Huganir’s lab demonstrates that the reversible and bidirectional changes associated with LTP and LTD require protein kinase A (PKA), calcium/calmodulin-dependent protein kinase II (CaMKII) and PP1/2A [2]. CaMKII increases phosphorylation of the a-amino-3-hydroxy-5-methyl-4-isoxazolepropionic acid (AMPA) receptor GluR1 subunit on Ser 831 in naïve synapses, resulting in LTP. In contrast, protein phosphatases (including PP1/2A) dephosphorylate Ser 831, reversing the LTP to the naïve state. Importantly, low-frequency stimulation can activate protein phosphatases (including PP1/2A). Dephosphorylation of GluR1 Ser 845 in naïve synapses results in LTD, and PKA can phosphorylate Ser 845. PP1 not only regulates the expression of LTP and LTD through changing the morphology and maturation of spines in hippocampal CA1 neurons [3] but also has the ability to depress synaptic transmission at basal activity levels to affect memory processes [4]. PP2B also participates in memory formation [5].

PP2A, as a major member of the PPPs in the serine/threonine phosphatase family, participates in a wide range of essential signalling pathways and neurodevelopment [6]. PP2A has recently been shown to act as a key switch in the regulation of Alzheimer disease [7]. Inhibition of PP2A was reported to induce hyperphosphorylation of microtubule-associated protein tau and produce memory consolidation deficits in adult rats [8]. In addition, an electrophysiological study of memory updating demonstrated that older adults show a decline in their memory updating ability [9]. Mucic et al. found that PP2A participates in fear memory. They screened almost 800 hippocampal protein kinases and phosphatases and found that PP2A was directly linked to the retrieval phase of contextual fear conditioning [10]. We hypothesized that PP2A is a key player in fear memory extinguishing. To address this issue, we used transgenic technology to specifically knockout PP2A in the hippocampal CA1 area and adopted contextual fear conditioning (cFC) to test the influence of PP2A deficiency on hippocampus-dependent contextual fear memory formation, encoding and maintenance and the regular mechanism.

## Materials and methods

### Animals

The *PP2A*^*flox/flox*^ wild-type mice were provided by Xiang Gao’s lab [11]. The *Tg (Camk2a-cre) T29–2Stl* mice, which mediate *Cre/loxP* recombination predominantly in CA1 pyramidal cells, were a kind gift from Prof. Tsai L-H (Picower Institute for Learning and Memory, MIT, USA). We crossed the *PP2A*^*f/f*^ control (Cont) mice with *T29–2 Cre* transgenic mice [12, 13] to generate the hippocampal CA1-specific PP2A conditional knockout (CKO) mice. We used 8- to 10-week-old mice in the behavioural experiments (male mice, *N* = 54/group), PCR (male and female mice, *N* = 3/group), western blot (male and female mice, N = 3/group) and immunohistochemistry (male and female mice, *N* = 4/group) and 4- to 6-week-old mice in the electrophysiology recording experiments (male and female mice, Cont group: *N* = 24, CKO group: *N* = 20). Separate animals were used for the behavioural tests, biochemical experiments and electrophysiology recordings. All animals were given ad libitum access to food and water and were housed in groups with males and females apart under a 12-h light/dark cycle. All animal experiments were performed in accordance with the recommendations of the Experimental Animal Ethics Committee at the Nanjing Medical University.

### Polymerase chain reaction (PCR)

To identify the genotype of the mice, we collected toes form 7–9 days old mice. The protocol of PCR was performed as described previously [11]. Briefly, to identify the genotype, the toe was collected from mice (before 10-day-old) to isolate DNA for PCR. The sequences of primers are listed as follows:

loxP-Forward primer: 5′ > TAGCCCATGCCTTTAATCTCAGAGC< 3′.

loxP-Reverse primer: 5′ > CACTCGTCGTAGAACCCATAAACC< 3′.

Cre-Forward primer: 5′ > TGCCACGACCAAGTGACAGCAATG< 3′.

Cre-Reverse primer: 5′ > ACCAGAGACGGAAATCCATCGCTC< 3′.

During the procedures of PCR, we first denatured DNA at 95 °C for 5 min, and then denaturing at 94 °C for 30 s. The step of annealing was at 58 °C for 30 s and extending at 72 °C for 1 min. After repeating for 35 more times, we extended DNA strands at 72 °C for 5 min. Then, the PCR products were analyzed by 1% agarose (BA0047, Nanjing best biological technology Co.,Ltd) gel electrophoresis and developed under ultraviolet light using Gel Image System (Tanon-2500, Shanghai, Tianneng Technology Corporation).

### Western blot analysis

To confirm the specific PP2A knockout, the brain (except for the olfactory bulb and cerebellum), including the hippocampal CA1 area, was collected from Cont and CKO mice. Coronal hippocampal slices were prepared at 500-μm thickness using a Leica VT1000S vibratome (Leica Instruments Ltd., Wetzlar, Germany) in ice-cold oxygenated (95% O_2_/5% CO_2_) cutting ACSF containing (in mM) 75 sucrose, 87 NaCl, 2.5 KCL, 1.25 NaH_2_PO_4_, 21.4 NaHCO_3_, 0.5 CaCl_2_, 7 MgCl_2_, 1.3 ascorbic acid and 20 D-glucose (pH 7.2–7.4). The hippocampal CA1 area was dissected with surgical blades and forceps. Lysates (50 mM MOPS, 100 mM KCl, 50 mM NaF, 20 mM NaPPi, 20 mM Glycerd-P, 320 mM Sucrose, 0.2 mM DTT, 1 mM EDTA, 1 mM EGTA, 0.5 mM MgCl_2_, 1 mM NaVO_4_, half of a protease inhibitor tablet in 10 ml) were incubated on ice and cleared with an 8000-rpm spin for 15 min, and protein content was quantified (BCA protein assay, Thermo Scientific). Four hundred micrograms of protein was diluted with 5× loading buffer consisting of the following: 250 Mm Tris pH 6.8, 10% SDS (w/v), 0.5% bromophenol blue (w/v), 50% glycerol (v/v), 5% β-mercaptoethanol. Samples were boiled at 95 °C for 10 min and resolved on a 10% SDS-polyacrylamide gel with 8% stacking gels using Laemmli buffer. Proteins were transferred by electrophoresis using tris-glycine wet transfer onto PVDF membranes (Millipore, 0.45 μm) for 1 h on ice. After blocking with blocking buffer (5% non-fat dry milk/0.1% Tween-20/TBS) for 1 h, membranes were probed with the anti-PP2A C subunit antibody (#2038, Cell Signaling Technology, 1:3000) and Tubulin β polyclonal antibody (AP0064, Bioworld, 1:1000) at 4 °C overnight. Membranes were washed three times using 0.1% Tween-20/TBS and incubated with a goat anti-rabbit IgG (H + L) HRP-linked antibody (BS13278, Bioworld, 1:8000) for 1 h at room temperature. Membranes were washed again and developed using Western Lightning Gel Imaging System (Tanon 2500, Shanghai, Tianneng Technology Corporation).

### Immunohistochemistry

The mice were perfused with 4% paraformaldehyde in phosphate-buffered saline, and the brain was dissected and placed in sucrose solution. After cryoprotection using a 15 and 30% sucrose gradient, coronal hippocampal slices were prepared at 25-μm thickness using a freezing microtome (CM-1950, LEICA). To confirm the efficiency of the specific PP2A knockout and the effect on the development of neurons and neurogliocytes in the hippocampal CA1 area, the slices were incubated in primary antibody overnight at 4 °C. After incubation with the secondary antibody for 2 h and DAPI (10,236,276,001, Roche, 1 μg/ml) for 15 min at room temperature, the samples were examined by using confocal laser microscopy (FV-1000, OLYMPUS). The antibodies and dilutions were as follows: PP2A C subunit antibody (#2038, Cell Signaling Technology, 1:250), anti-NeuN rabbit polyclonal antibody (ABN78, Millipore, 1:500), goat anti-rabbit IgG (H + L) Cy3 (BS10007, Bioworld Technology, 1:400) and anti-glial fibrillary acidic protein (GFAP) antibody, and clone GA5 (MAB3402, Millipore, 1:500).

### Behavioural experiments

#### Open field test

Locomotor activity and anxiety responses of rodents can be tested using an open field test [14, 15]. The open field apparatus (50 × 50 cm, Shanghai Xinruan Informatlon Technology Co. Ltd., Shanghai) was divided into 16 compartments in the ANY-Maze software (Stoelting, Illinois), and the 4 in the middle were defined as the centre area. The mice (*N* = 12/group) were individually placed in one corner of the open field apparatus and allowed to explore freely for 10 min. Mouse movement was tracked by ANY-Maze tracking software (Stoelting, Illinois). The distance moved in the apparatus every 2 min and total time spent in the centre area were recorded.

#### Forced swim test

The forced swim test was conducted according to the Porsolt protocol [16]. Mice (Cont group: *N* = 14, CKO group: *N* = 11) were forced to swim for 6 min in a big glass cylinder filled with water at 25 ± 1 °C. The immobility time during last 4 min was recorded to evaluate depression-like behaviour.

#### Prepulse inhibition (PPI) of startle reflex test

The PPI of the acoustic startle response was tested as described previously [17]. The mice (*N* = 12/group) were habituated to the chamber with a white-noise background (70 dB) for 5 min. Each test consisted of 80 trials with 6 null trials, 68 prepulse-pulse trials, and 6 pulse-alone trials. The average intertrial interval was 15 s (range from 10 to 20 s). Null trials consisted of a 40-ms burst of a 120-dB stimulus. Prepulse-pulse trials included 7 types of trials presented randomly, including a 40-ms burst of a 120-dB single stimulus, a 40-ms prepulse stimulus that was 74, 82, or 90 dB and three prepulse stimuli followed 100 ms later by a 120-dB stimulus. The test terminated with pulse-alone trials using the same protocol as used for the null trials. PPI responses were calculated as % PPI = [1– (prepulse trials/startle-only trials)] × 100%.

#### Object recognition test

The object recognition test was performed as described previously in the literature [18, 19]. Briefly, mice (Cont group: *N* = 8, CKO group: *N* = 10) were habituated in the empty open field for 5 min each day for 1 week. Two identical 150-ml bottles were placed in their cages to serve as “old objects”. After the habituation phase, two identical old objects were placed in the open field at an equal distance from the mice. The familiarization session lasted for 2 days during which mice were placed in the open field for 5 min four times a day. Then, the trained mice were divided into short-term memory (STM) and long-term memory (LTM) groups. At the beginning of the test session, all animals were allowed to explore the old objects for 5 min. One hour later in the STM group and 24 h later in the LTM group, the animals were placed in the open field with one old and one new object. The test session lasted 10 min. The time the mouse spent sniffing (sniff time) the old and new objects was recorded by using ANY-Maze tracking software. The memory index was used to evaluate the memory function of mice: Memory Index = (Sniff time of new object – Sniff time of old object)/(Sniff time of new object + Sniff time of old object) × 100%.

#### Contextual fear conditioning (cFC)

The experimental protocol was modified from work published previously [20]. The animals (Cont group: *N* = 8, CKO group: *N* = 9) were placed in the chambers for 3 min. After habituation, three consecutive foot shocks of 0.7 mA lasting for 2 s at 2 min intervals were administered to form the conditioned fear memory. On the second day, all animals were returned to the same chamber, and freezing was automatically recorded using FRAMEFREEZE software (Coulbourn Instruments) for 3 min. They were then removed from the chamber and returned to their home cages. One hour later, the animals were put back into the chamber for 21 min without receiving foot shocks and then returned to their home cages for 21 min. This extinction phase process was repeated three times. Freezing was recorded for 3 min 24 h, 48 h, and 72 h after the extinction phase.

### Electrophysiological analysis

Electrophysiological recordings were performed as previously described in Yang et al [21] Horizontal hippocampal slices were prepared at 350-μm thickness using a Leica VT1000S vibratome (Leica Instruments Ltd., Wetzlar, Germany) in ice-cold oxygenated (95% O_2_/5% CO_2_) cutting artificial cerebrospinal fluid (ACSF) containing (in mM) 75 sucrose, 87 NaCl, 2.5 KCL, 1.25 NaH_2_PO_4_, 21.4 NaHCO_3_, 0.5 CaCl_2_, 7 MgCl_2_, 1.3 ascorbic acid and 20 D-glucose (pH 7.2–7.4). Slices were transferred to a holding chamber and incubated for 60 min at 32 °C submerged in oxygenated (95% O_2_/5% CO_2_) recording ACSF containing (in mM) 119 NaCl, 2.5 KCl, 1 NaH_2_PO_4_, 26.2 NaHCO_3_, 2.5 CaCl_2_, 1.3 MgSO_4_ and 11 D-glucose (pH 7.2–7.4). The slices were then incubated at room temperature for at least 1 hour before recording.

The stimulator was placed in the Schaffer collateral/commissural pathway. Recording electrodes (resistance, 1–4 MΩ) were pulled from borosilicate glass capillary tubes (1.5-mm outer diameter, 0.86-mm inner diameter, World Precision Instruments) using a Brown-Flaming micropipette puller (P-97; Sutter Instruments Company) and filled with recording ACSF. Field excitatory postsynaptic potentials (fEPSPs) in the hippocampal CA1 area were recorded. We chose the slices whose maximal fEPSP amplitude was at least 0.7 mV, and the stimulation intensity was adjusted so that baseline fEPSPs were recorded at 40% of the maximal amplitude. Input-output data were collected by varying the intensities of seven stimuli applied to the CA1 area. Paired-pulse facilitation induced by paired-pulse stimulation (inter-pulse intervals were 10 ms, 20 ms, 50 ms, 100 ms and 200 ms) were evoked every 30 s. After a 10-min stable baseline, LTP was induced by two theta burst stimulations (TBSs) separated by 20 s (5 trains at 5 Hz with each train including 4 pulses at 100 Hz) or 50-Hz high-frequency stimulation (HFS) (5 trains of 1-s stimulation at 50 Hz with 200-ms inter-train intervals) followed by 40 min of fEPSP recording. To investigate the changes in depotentiation in Cont and CKO mice, we first used four TBSs separated by 20 s to induce a saturated LTP. After 45 min, we used low-frequency stimulation (LFS) (900 trains of 15 min stimulation at 1 Hz) to induce depotentiation [22, 23].

Changes in LTD expression in Cont and CKO mice were also detected. After a 15-min stable baseline, LTD induced by LFS was recorded for 45 min. Pharmacological treatment was used to further verify the role of PP2A in LTD. A stock solution of PP2A inhibitor okadaic acid (OA, Sigma) dissolved in 0.1% dimethyl sulphoxide (DMSO) was prepared and stored at − 20 °C [2]. The stock OA solution was mixed with freshly made recording ACSF to a final concentration of 25 nM [24]. Before electrophysiological recording, we incubated the slices from Cont mice in OA solution for 30 min, and during LTD recording, the slices were maintained under OA treatment.

The LTP and LTD magnitude was calculated from the average of the last 10 min of recording and reported as the (%) Mean ± SEM of baseline fEPSP slope.

### Statistical analysis

Data were analysed using SPSS 19.0 (SPSS, Inc., Chicago, IL, USA), and illustrations were created using Origin 8.5 (Electronic Arts Inc., California, USA). Differences in the behaviour tests between Cont and CKO mice were tested for statistical significance using an independent *t* test. To further assess the effect of PP2A on memory extinction, we analysed the cFC data, input-output curves, paired-pulse stimulation and the last 10 min of the fEPSP slope after LTP or LTD of the Schaffer Collateral-CA1 pathway using a repeated measures ANOVA. Data were reported as the mean ± SEM. The significance level for all tests was set at *p* < 0.05.

## Results

### Generation of the hippocampal CA1-specific PP2A knock-out mice

**Fig. 1:**
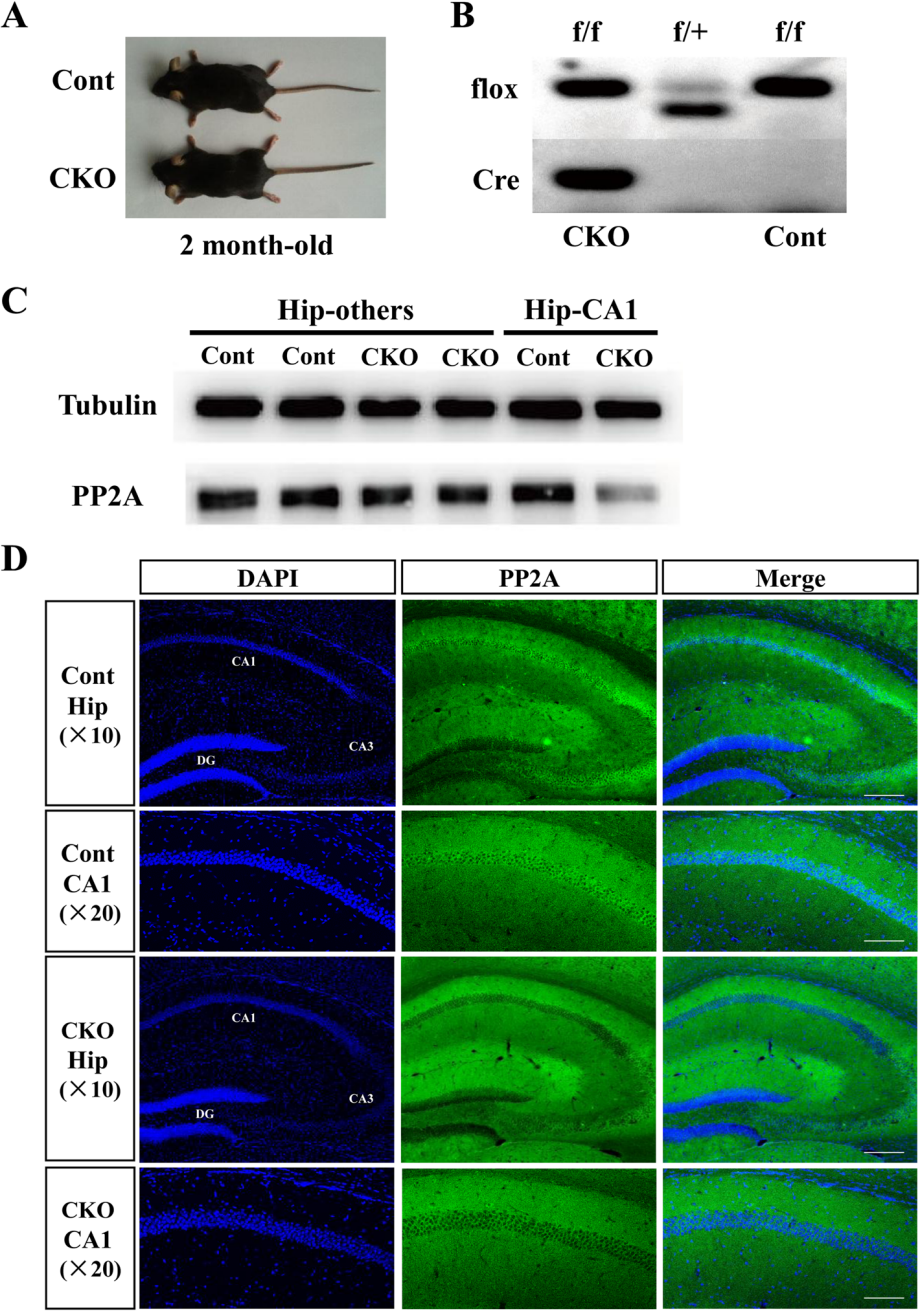
PP2A-specific knockout efficiency. **a** Photo of Cont and CKO mice at 2 months old. **b** The genotype of the mice. The mouse who had both two kinds of sequences was identified as conditioned knockout (CKO), and the genotype of those had loxP sequences only was identified as control (Cont). **c** Western blot analysis of hippocampus tissue from Cont and CKO mice. There was no difference in other tissue without CA1 area of hippocampus (Hip-others) in both genotype of mice. But PP2A CKO mice showed a significance decrease expression of PP2A in CA1 region of hippocampus (Hip-CA1). **d** Immunohistochemistry with the PP2A C subunit antibody to confirm specific knockout of PP2A within the CA1 region of the hippocampus in CKO mice. Scale bar of (× 10) represents 50 μm; scale bar of (× 20) represents 100 μm

Since *PP2A*: nestin-cre mice generally died after birth, we generated the mice lacking *PP2A* specifically in hippocampus neurons by mating the *PP2A*^flox/flox^ mice [11] with the Cre line T29–2, in which Cre is highly expressed in CA1 pyramidal neurons of the hippocampus [12, 13]. Mice with homozygous deletion of *PP2A* in the hippocampus were born at expected Mendelian ratios and showed normal body weight (Fig. 1a, 2-month-old weight of PP2A CKO mice and Cont mice: 23.7 ± 0.5 g and 23.1 ± 0.6 g). Successful deletion of PP2A in the brain of mutant mice was validated by PCR (Fig. 1b) and Western blot analysis (Fig. 1c). As shown in Fig. 1c, PP2A protein levels were dramatically reduced in the hippocampus CA1 of 2.5-month PP2A CKO mice. Immunofluorescence analyses further showed that PP2A was successfully knocked out in hippocampal CA1 neurons (Fig. 1d).

### PP2A CKO mice displayed normal locomotion or exploratory activity

**Fig. 2:**
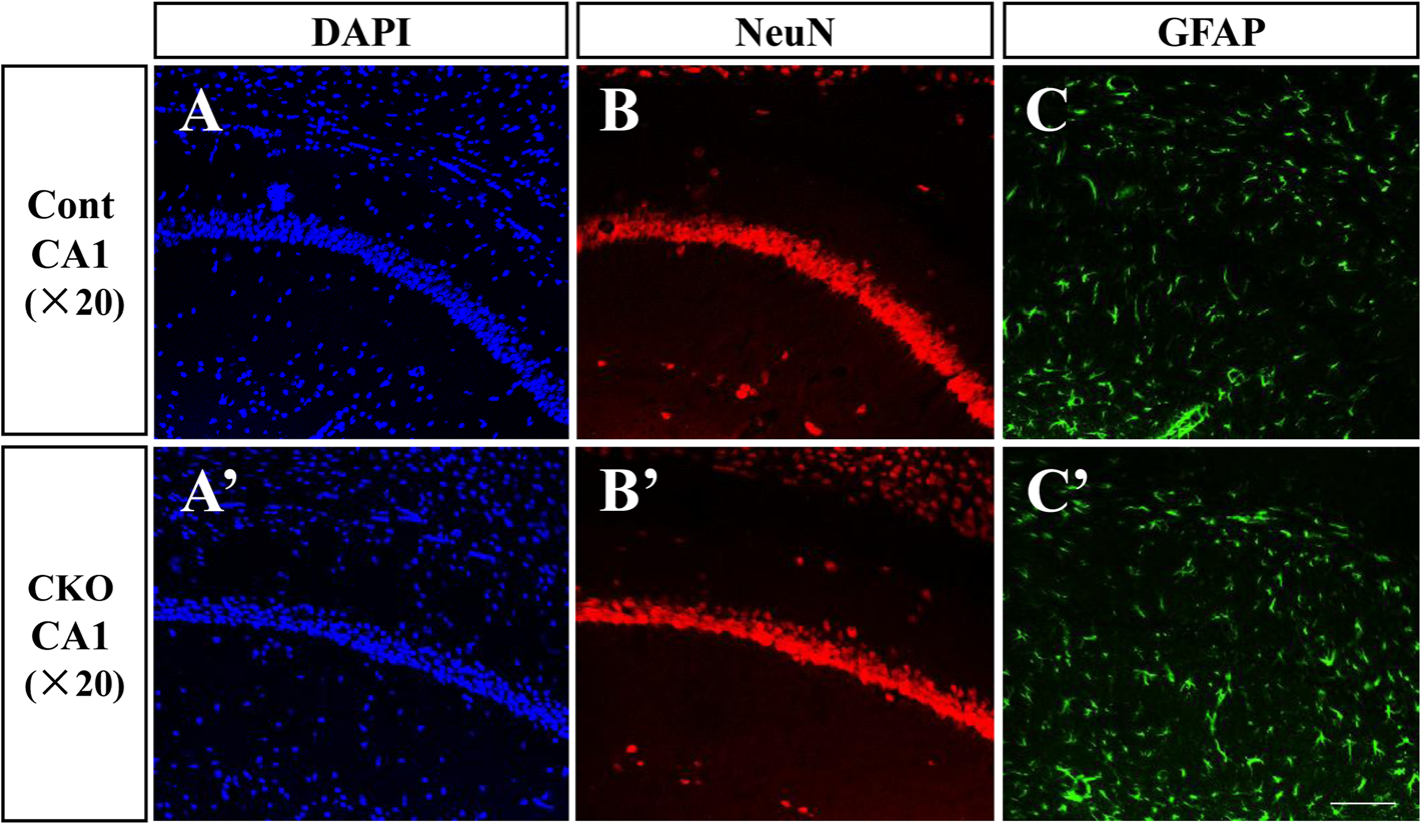
Deficit of PP2A in CA1 had no effects on the development of the neural system. Immunohistochemistry of the hippocampal CA1 region with the DAPI, anti-NeuN mouse and anti-GFAP rabbit antibodies in Cont mice (**A**-**C**) and CKO mice (**A’-C’**). Scale bar represents 100 μm

**Fig. 3:**
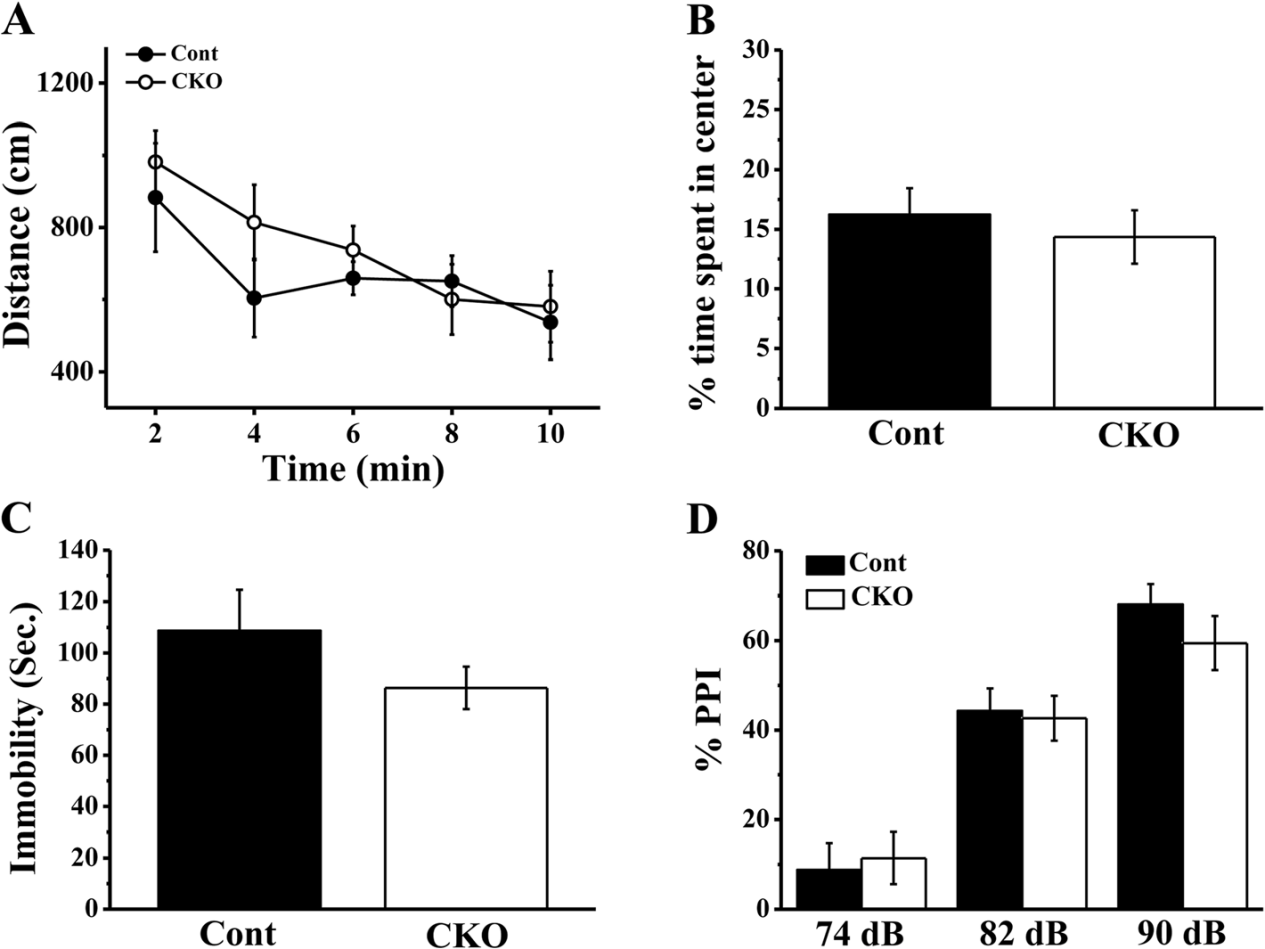
Locomotion activity and emotional related behaviours in PP2A CKO mice were almost not affected. **a** In the open field test, the distance moved in the apparatus every 2 min of Cont and CKO mice were the same (*N* = 12/group). **b** Both genotypes spent the same amount of time in the centre area of open field box (*N* = 12/group). **c** During the forced swim test, the immobility time of Cont (*N* = 14) and CKO mice (*N* = 11) had no significance difference. **d** The PPI responses with 74-dB, 82-dB and 90-dB pulses were the same for Cont and CKO mice (*N* = 12/group)

As mentioned in the previous data, the T29-Cre expression would spread to other brain regions in older (4-month-old) mice, while it was relatively specific to area CA1 in young mice (2–3.5 months old). Therefore, we used 8- to 10-week-old mice to perform all tests. The morphology of the neurocytes and neurogliocytes in the hippocampal CA1 area was unaffected by the conditional knockout (Fig. 2). To examine whether conditional knockout PP2A affected basic behaviour, we used several behavioural tests to assess locomotion, depression and schizophrenia-like behaviours of the CKO mice. In the open field test, we found travelled distance every 2 min and time spent in the central area of the open field were the same between Cont and CKO mice (Fig. 3a and b). Depression and schizophrenia-like behaviours were tested using the forced swim test and the PPI test. The immobility time of the CKO mice was a little shorter than that of the Cont mice in the forced swim test but no significance (Fig. 3c). In addition, there were no differences between Cont and CKO mice in the PPI test (Fig. 3d). These results suggested that PP2A deficit in hippocampual CA1 did not affect the basic behaviours and exploration abilities of the mice.

### Mice lacking PP2A in the hippocampal CA1 area had impaired memory extinction

**Fig. 4:**
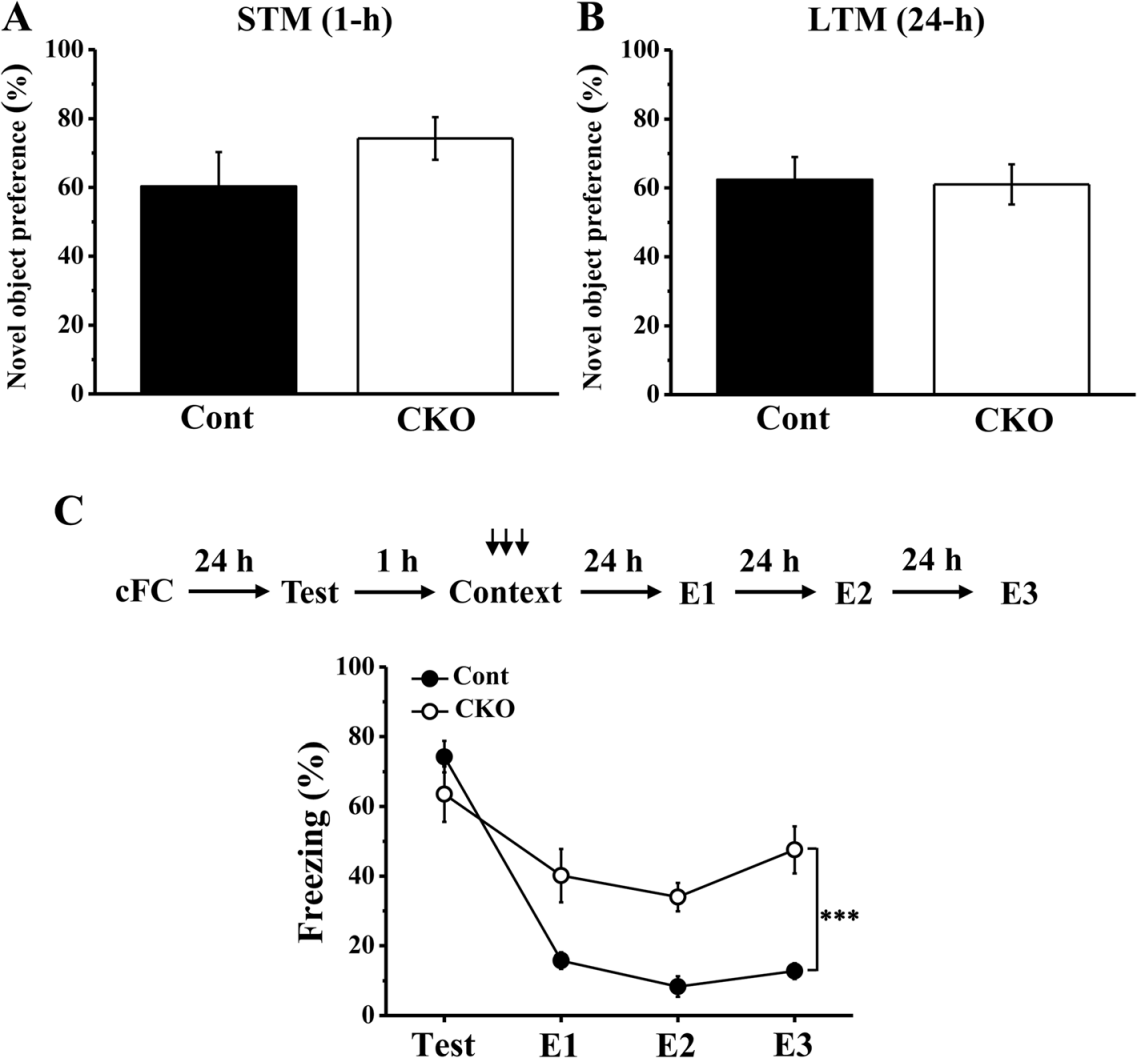
PP2A CKO mice had normal abilities of memory formation but an impairment in memory extinction. **a**, **b** In the object recognition test, CKO mice formed normal STM and LTM (Cont group: *N* = 8, CKO group: *N* = 10). **c** CKO mice (*N* = 9) and Cont mice (*N* = 8) formed contextual fear memory after three training trials. However, the freezing level of CKO mice was significantly higher than that of Cont mice over the 3 days of the cFC extinction phase. *** *p* < 0.001

Hippocampus plays a very important role in learning and memory. To investigate whether conditional PP2A knockout in the hippocampal CA1 area affected learning and memory, we tested STM and LTM using the novel object recognition test and found that there was no difference between the Cont and CKO mice (Fig. 4a and b). These results demonstrated that PP2A deficiency did not affect either short-term nor long-term memory formation. To further test whether PP2A CKO mice had normal ability of memory extinction, we used cFC training to evaluate the formation phase and extinction phase of memory. After three consecutive foot shocks, we found no significant difference between the two groups, both which showing a high level of freezing (Fig. 4c). During the cFC extinction phase, the freezing level of the CKO mice became significantly higher than that of the Cont mice (F_3, 45_ = 12.557, *p* < 0.001). These data indicated that conditional PP2A knockout in the hippocampal CA1 area had no effect on memory formation but impaired extinction.

### PP2A deficiency impaired basic synaptic transmission and synaptic plasticity

**Fig. 5:**
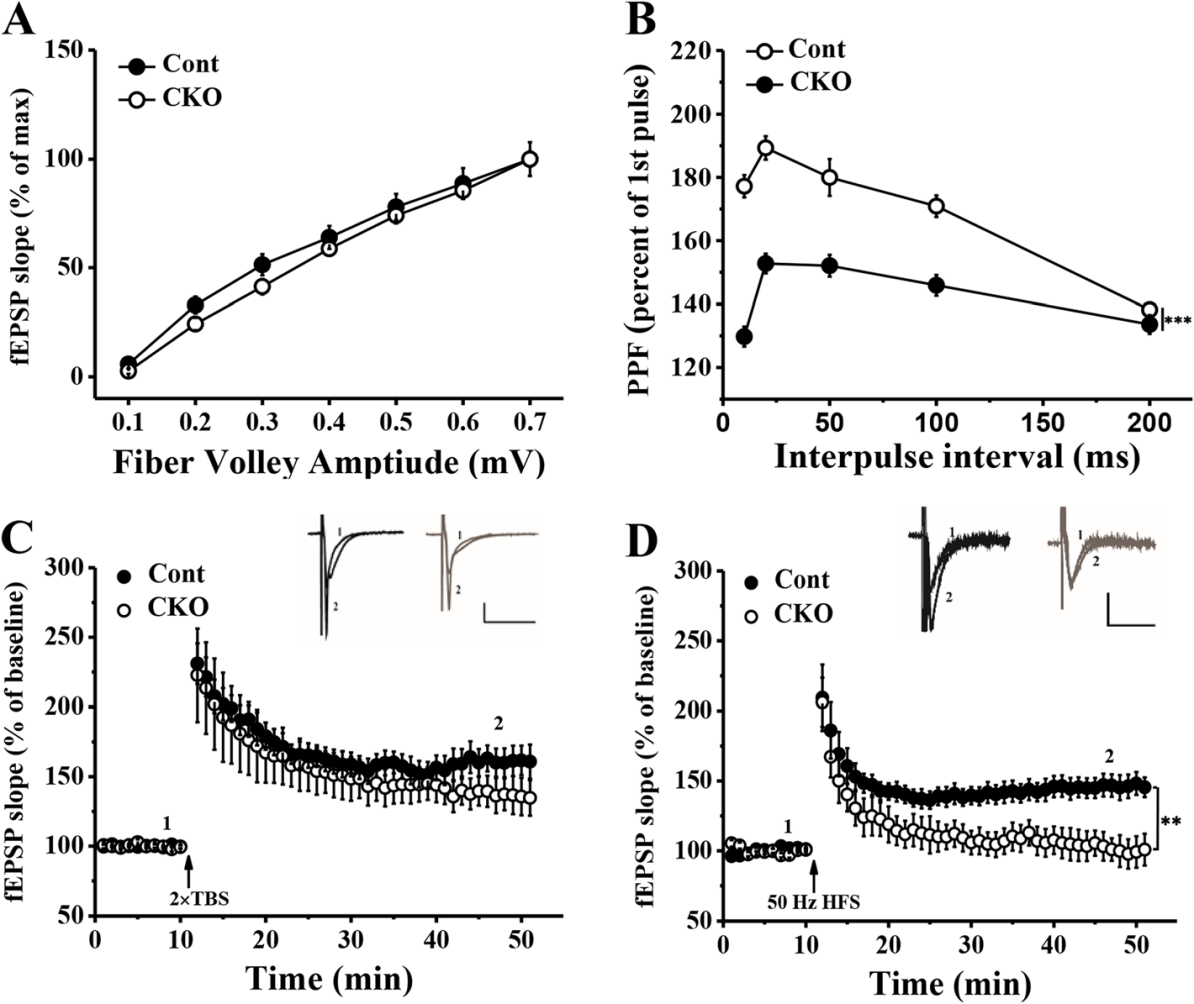
Change in basic synapse transmission and LTP in the hippocampus of PP2A CKO mice. **a** Input-output curves for the CA1 dendritic fEPSP slope evoked by Schaffer commissural fibre stimulation in hippocampal slices from Cont and CKO mice (*N* = 3/group, *n* = 9/group). **b** The basis of synaptic transmission capacity was significantly lower in CKO mice than in Cont mice (*N* = 5/group, *n* = 18/group). **c**, **d** LTP could be evoked by 2 × TBS in both Cont and CKO mice (C, *N* = 3/group, *n* = 6/group). However, LTP induced by 50-Hz HFS was impaired in CKO mice (D, *N* = 3/group, *n* = 6/group). Vertical scale bar represents 0.2 mV; horizontal scale bar represents 50 ms. ** *p* < 0.01, *** p < 0.001

**Fig. 6:**
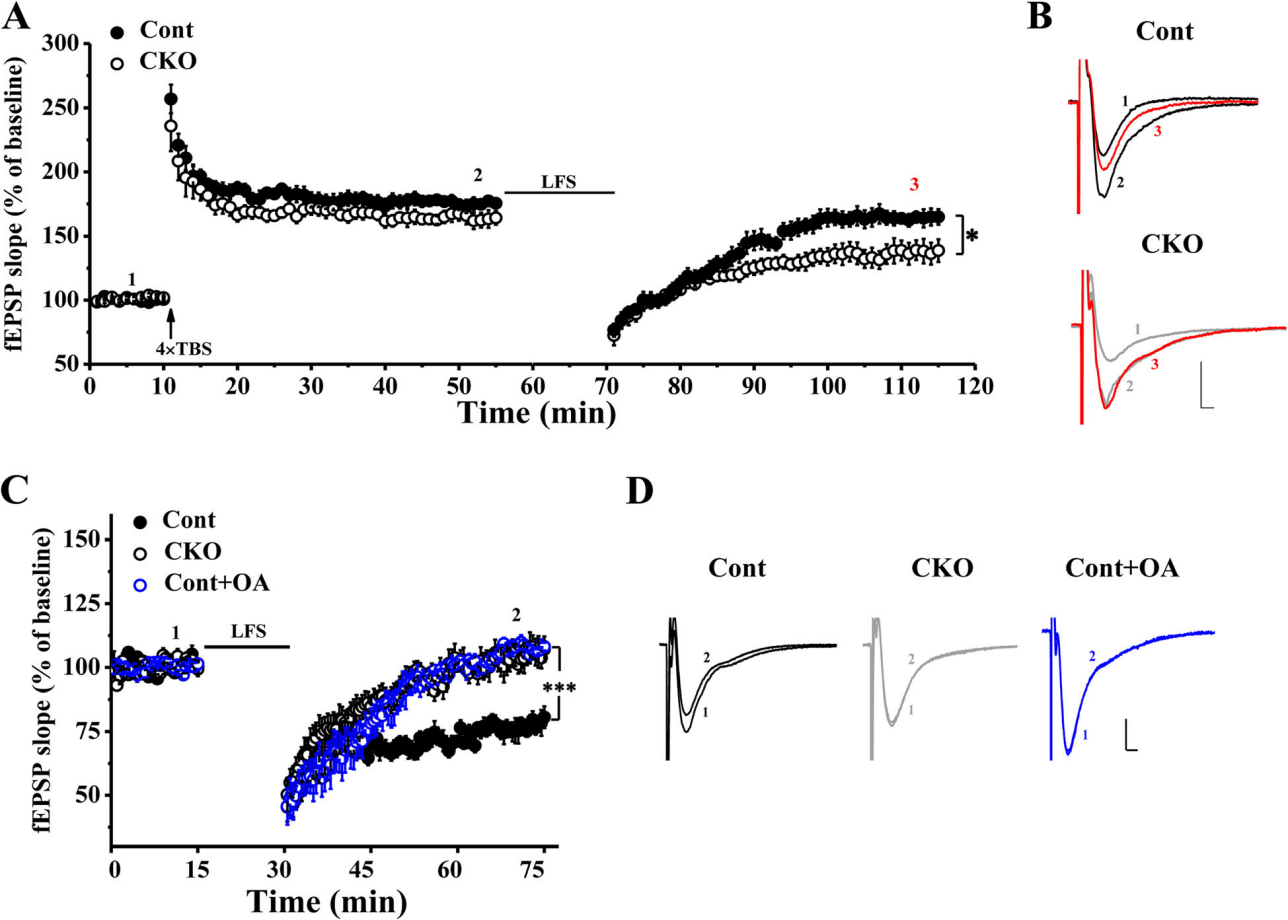
Depotentiation and LTD of CKO mice was impaired. **a** Stable LTP was produced by 4 × TBS in both Cont and CKO mice, but LFS did not induce a reversal of LTP in CKO mice (*N* = 3/group, *n* = 7/group). **b** Waves of the last 10 min on the phases of baseline (1, black), LTP induced by 4 × TBS (2, black) and reversal LTP induced by LFS (3, red). The line of Cont group was black and that of CKO group was gray. **c** LTD could be induced by LFS in Cont mice but not in CKO mice. Meanwhile, LFS-induced LTD was significantly blocked by the PP2A inhibitor (Cont group: *N* = 3, *n* = 7, CKO group: *N* = 3, *n* = 7, Cont+OA group: *N* = 4, *n* = 6). **d** Waves of the last 10 min on the phases of baseline (1, black) and LTD induced by LFS (2, black). The line of Cont group was black and that of CKO group and Cont+OA group were gray and blue. Vertical scale bar represents 0.2 mV; horizontal scale bar represents 50 ms. * *p* < 0.05, *** p < 0.001

The underlying cellular mechanism of learning and memory is believed to be synaptic plasticity. Thus, next we examined whether synaptic plasticity in the hippocampus was affected by PP2A knockout. As shown in Fig. 5a, the input-output curve slopes were not altered by PP2A deficiency. Furthermore, the synaptic efficacy was tested by paired-pulse facilitation (PPF). The CKO mice showed a significant decrease in PPF at inter-pulse intervals of 10 ms, 20 ms, 50 ms and 100 ms (F_4, 136_ = 14.310, *p* < 0.001) (Fig. 5b). Considering that the attenuation of PPF is associated with synaptic potentiation, we then measured LTP induction by 2 × TBS but found no differences between Cont and CKO mice (Fig. 5c). However, the induction of LTP by 50-Hz HFS was significantly impaired in the hippocampus slice from the conditional PP2A knockout mice (Fig. 5d, F_1, 10_ = 11.487, *p* < 0.01). To investigate the cellular mechanisms of memory flexibility, we used the depotentiation protocol in the CA1 region of hippocampus slices from the Cont or CKO mice. Depotentiation is considered a model to measure the ability for LTP reversal. We found that depotentiation was inhibited in CKO mice compared with that in Cont mice (Fig. 6a, F_1, 12_ = 7.649, *p* < 0.05). Meanwhile, the LFS (1 Hz, 15 min) stimulation could induced LTD in the hippocampus slices from Cont mice but not that from CKO mice. Similarly, acute application of the PP2A antagonist OA in Cont mice inhibited the induction of LTD (Fig. 6c, F_2, 17_ = 23.024, *p* < 0.001). In summary, the results suggested that lacking PP2A in the hippocampal CA1 area impaired synaptic efficacy, 50-Hz HFS-induced LTP, depotentiation, and LFS-induced LTD. Furthermore, this decreased synaptic efficacy in the hippocampal CA1 area may contribute to the impaired memory extinguishing ability in PP2A CKO mice.

## Discussion

Our data provide insights into the function of PP2A in memory extinction. To examine this hypothesis, we chose CKO mice with PP2A knocked out in the hippocampal CA1 area by crossing *PP2A*^*f/f*^ mice with Cre transgenic mice. Immunobloting and immunofluorescence analysis showed less PP2A expression in the CA1 region of the CKO mice. Meanwhile, a lack of PP2A in the CA1 region of the hippocampus had no effect on the morphology of hippocampal neurocytes or neurogliocytes in adult mice. The PP2A CKO mice showed no movement disorders and normal basic behaviours compared with that of Cont mice. Although PP2A deficiency did not influence short-term or long-term memories in the object recognition test, our data on contextual fear memory show that PP2A levels were strongly associated with memory extinction.

Declarative memory has been studied extensively using animal models such as new object recognition and conditioned fear memory tests. Fear memory helps animals detect and avoid previously encountered threats. One of the useful paradigms for studying different phases of hippocampal memory in rodents is the Pavlovian fear conditioning test, in which a conditioned stimulus (CS), such as a different background environment, is paired with an aversive unconditioned stimulus (US), such as a weak electric shock. After several CS-US pairings, the background environment elicits the freezing behavioural fear response. However, repeating the CS in the absence of the US (CS–no US) causes the conditioned responses to end [25, 26]. As early as the beginning of the twentieth century, Pavlov demonstrated that extinction did not erase the conditioned memory but inhibited the conditioned response. In other words, extinction is not a loss of memory itself but a deficit in retrieval [27]. Putting animals in a CS–no US environment initiates a new learning process that inhibits the process of conditioned fear memory that has already formed [28]. Using the fear conditioning animal model, Preethi et al. [29] shows that contextual fear memory is improved by modulating PP2A levels with an extract of Bacopa monniera. Consistent with previous work, our findings suggest that loss of PP2A in the hippocampal CA1 area does not affect the process of conditioned fear memory formation. This finding is consistent with our observations that the CKO mice did not show appreciable deficits in short-term and long-term object recognition tests. But the CKO mice were able to retrieve the fear memory 3 days after the event. Thus, the updating ability of memory was impaired in the CKO mice.

Properties of synaptic plasticity suggest a role in memory [30]. Paired-pulse facilitation (PPF) is one of forms of short-term synaptic plasticity, which is important for long-term forms of plasticity induction. It is primarily associated with increased presynaptic Ca^2+^ concentration leading to a greater transmitter release [31]. And weaker paired-pulse facilitation suggests high neurotransmitter release probability [32, 33]. Whereas, PPF or neural facilitation of different types of synapses had different Ca^2+^ use-dependent manners. Recent study showed that there was no use-dependent increase in calcium entry in Schaffer collateral synapses between hippocampal CA3 and CA1 pyramidal cells [34]. Here, we found the magnitude of PPF was decreased in slices from PP2A CKO mice suggesting that the probability of neurotransmitter release was increased. But the efficacy of synaptic transmission, assayed with the input-output cures remained unchanged. Here, the deletion of PP2A was selectively in postsynaptic CA1 neurons. Although the mechanism of neural facilitation is mainly presynaptic, postsynaptic contributions cannot be excluded. Previous studies showed that calcium permeable AMPA receptors involved in short-term enhancement of synaptic strength [35, 36]. Meanwhile, PP2A had no effect on Ca^2+^-induced neurotransmitter release [37]. Thus, PP2A conditioned knockout in hippocampal CA1 area had no effect on input-output curves, which is a calcium-dependent process [38, 39]. And PP2A might regulate facilitation by regulating the dehosphorylation of AMPA receptors. It needs to be proved by subsequent experiments.

LTP is one of the prime candidates for learning and memory. Since its discovery [40, 41], the cellular changes associated with LTP have been widely investigated [42]. LTP can be induced by high-frequency synaptic stimulation, which leads to the influx of Ca^2+^ through the N-methyl D-aspartate (NMDA) receptor and the activation of CaMKII. PP2A is required for the late phase of LTP [43] and modulates learning and memory via regulation of CaMKII, an important mediator of synaptic plasticity [44]. Here, we investigated LTP in CKO mice using a TBS protocol and found that LTP induction was normal. Because the mechanism of LTP induction between stimulation protocols is different, we also tested LTP induction using HFS. Hernandez et al. [45] demonstrated a linear relationship between the number of pulses applied during stimulation and the intensity of LTP induction, regardless of TBS or HFS. Thus, we chose 50 Hz HFS to avoid inducing saturated LTP. Interestingly, this protocol was unable to induce LTP in CKO mice. Recent study shows that TBS- and HFS-induced LTP rely on different intracellular pathways to trigger actin polymerization [46]. TBS decreased calpain-1-mediated suprachiasmatic nucleus circadian oscillatory protein (SCOP) and increased phosphorylated extracellular regulated kinase (ERK), but HFS increased PKA phosphorylation. Consistent with previous results that PP1/2A was required in LTP and LTD induction [2], further investigation on the molecular mechanism are required. Another important candidate for the molecular basis of learning and memory is LTD, which may function to weaken previous memory traces [47]. LTD is induced by low-frequency synaptic stimulation, which activates protein phosphatases by leading to moderate, prolonged increases in Ca^2+^ levels. Facilitation of LTD is necessary for the acquisition and updating of memory [48]. PP2A is required for LTD [2, 49, 50], and a PP2A inhibitor can block LFS-induced LTD. Okadaic acid (OA) is a potent inhibitor of PP1 and PP2A. After intracerebroventricularly injected OA into lateral ventricles, the fEPSP slope and population spike of rats in dentate gyrus (DG) neurons were attenuated [51]. As in previous studies, OA blocked the LTD induced by LFS in the Cont mice. As well as, LTD was impaired in the CKO mice.

Memory consolidation and retrieval are widely thought to be related to the reactivation of previously stored patterns of neural activity. LTP reversal or depotentiation is thought to be important in the acquisition of new information. Depotentiation reverses LTP, suggesting that the hippocampus is able to update a memory after the consolidation phase. A depotentiation in the hippocampal CA1 area has been reported to be induced by LSF after HFS induction [22, 52]. To determine whether the loss of PP2A in the hippocampal CA1 area affected depotentiation, we used four TBSs separated by 20 s to induce a saturated LTP. After 45 min, we used LFS to induce depotentiation in the CA1 region. We found that CKO mice did not exhibit depotentiation, further demonstrating that PP2A deletion results in an impaired ability to update memory. Depotentiation shares many similarities with LTD, but they are different. LTD is a basal synaptic response, whereas depotentiation is a reduction in synaptic strength previously increased by LTP [53]. Furhtermore, LTD but not depotentiation, is blocked by calcineurin inhibitors [52, 54, 55]. Evidence from Richard’s lab demonstrates that mouse lacks S845 can induce depotentiation but not LTD and S845 is critical for LTD expression [56]. But our electrophysiological results about LTD and depotentiation are consistent. LTD was impaired in the PP2A CKO mice, as well as, PP2A CKO mice did not exhibit depotentiation. These results are consistent with our behavioral results of PP2A CKO mice. These results imply that the dephosphorylation site of PP2A may not only S845.

In conclusion, the present study shows that PP2A deficiency does not affect memory formation, but the ability of memory extinction in conditional PP2A knockout mice is impaired. These observations indicate that PP2A is involved in the regulation of memory extinction. In addition, further studies of the precise molecular pathways of PP2A are still required.

## Abbreviations

ACSF: Artificial cerebrospinal fluid
AMPA: A-amino-3-hydroxy-5-methyl-4-isoxazolepropionic acid
CaMKII: Calcium/calmodulin-dependent protein kinase II
cFC: contextual fear conditioning
CKO: Conditional knockout
Cont: Control
CS: Conditioned stimulus
DG: Dentate gyrus
DMSO: Dimethyl sulphoxide
ERK: Extracellular regulated kinase
fEPSPs: field excitatory postsynaptic potentials
HFS: High-frequency stimulation
LFS: Low-frequency stimulation
LTD: Long-term depression
LTM: Long-term memory
LTP: Long-term potentiation
NMDA: N-methyl D-aspartate
OA: Okadaic acid
PCR: Polymerase chain reaction
PKA: Protein kinase A
PP1: Protein phosphatase 1
PP2A: Protein phosphatase 2A
PP2B: Protein phosphatase 2B
PPF: Paired-pulse facilitation
PPI: Prepulse inhibition
PPPs: Phosphoprotein phosphatases
SCOP: Suprachiasmatic nucleus circadian oscillatory protein
STM: Short-term memory
TBS: Theta burst stimulation
US: Unconditioned stimulus
